# Effects of epinephrine and vasopressin on end-tidal carbon dioxide tension and mean arterial blood pressure in out-of-hospital cardiopulmonary resuscitation: an observational study

**DOI:** 10.1186/cc5726

**Published:** 2007-03-21

**Authors:** Stefan Mally, Alina Jelatancev, Stefek Grmec

**Affiliations:** 1 Centre for Emergency Medicine Maribor, Ljubljanska 5, 2000 Maribor, Slovenia

## Abstract

**Introduction:**

Clinical data considering vasopressin as an equivalent option to epinephrine in cardiopulmonary resuscitation (CPR) are limited. The aim of this prehospital study was to assess whether the use of vasopressin during CPR contributes to higher end-tidal carbon dioxide and mean arterial blood pressure (MAP) levels and thus improves the survival rate and neurological outcome.

**Methods:**

Two treatment groups of resuscitated patients in cardiac arrest were compared: in the epinephrine group, patients received 1 mg of epinephrine intravenously every three minutes only; in the vasopressin/epinephrine group, patients received 40 units of arginine vasopressin intravenously only or followed by 1 mg of epinephrine every three minutes during CPR. Values of end-tidal carbon dioxide and MAP were recorded, and data were collected according to the Utstein style.

**Results:**

Five hundred and ninety-eight patients were included with no significant demographic or clinical differences between compared groups. Final end-tidal carbon dioxide values and average values of MAP in patients with restoration of pulse were significantly higher in the vasopressin/epinephrine group (*p *< 0.01). Initial (odds ratio [OR]: 18.65), average (OR: 2.86), and final (OR: 2.26) end-tidal carbon dioxide values as well as MAP at admission to the hospital (OR: 1.79) were associated with survival at 24 hours. Initial (OR: 1.61), average (OR: 1.47), and final (OR: 2.67) end-tidal carbon dioxide values as well as MAP (OR: 1.39) were associated with improved hospital discharge. In the vasopressin group, significantly more pulse restorations and a better rate of survival at 24 hours were observed (*p *< 0.05). Subgroup analysis of patients with initial asystole revealed a higher hospital discharge rate when vasopressin was used (*p *= 0.04). Neurological outcome in discharged patients was better in the vasopressin group (*p *= 0.04).

**Conclusion:**

End-tidal carbon dioxide and MAP are strong prognostic factors for the outcome of out-of-hospital cardiac arrest. Resuscitated patients treated with vasopressin alone or followed by epinephrine have higher average and final end-tidal carbon dioxide values as well as a higher MAP on admission to the hospital than patients treated with epinephrine only. This combination vasopressor therapy improves restoration of spontaneous circulation, short-term survival, and neurological outcome. In the subgroup of patients with initial asystole, it improves the hospital discharge rate.

## Introduction

Epinephrine (adrenaline) has been employed for cardiac resuscitation for more than a century, despite the knowledge that it can cause beta-mimetic complications [1-3]. Vasopressin is a potent vasopressor that could become a useful therapeutic alternative in the treatment of cardiac arrest because it has very little effect on pulmonary circulation and ventilation/perfusion mismatch [4-6]. Our previous study shows that vasopressin could become a better alternative to epinephrine [6]. Recent studies have shown that vasopressin is especially beneficial when combined with epinephrine during cardiopulmonary resuscitation (CPR) [1,7].

Several studies show a strong correlation between end-tidal carbon dioxide tension (pet_CO2_) and cardiac output, coronary perfusion pressure (CPP) and cerebral perfusion pressure, restoration of spontaneous circulation (ROSC), and hospital discharge [8-13]. In addition, clinical studies were performed to demonstrate the correlation between mean arterial blood pressure (MAP) and survival as well as the neurological outcome after CPR [14,15].

The aim of this prehospital study was to compare the values of pet_CO2 _and MAP in patients who suffered a cardiac arrest. They were divided in two groups; one was treated with epinephrine and the other with vasopressin. Our goal was to demonstrate that the use of vasopressin during CPR contributes to higher pet_CO2 _and MAP values and thus may have a beneficial impact on survival rate as well as on neurological outcome.

## Materials and methods

In this observational prospective study in the town of Maribor, Slovenia (approximately 200,000 inhabitants), we collected data from January 2000 to April 2006 with the approval of the ethical review board of the Ministry of Health. All emergency calls in this period which were classified as out-of-hospital cardiac arrest (OHCA) in adults older than 18 years and which were dispatched to the prehospital emergency unit were included. In the Centre for Emergency Medicine Maribor, we have two prehospital emergency teams, which are advanced life support (ALS) units of three members with adequately equipped road vehicles (an emergency physician and two registered nurses or medical technicians). ALS was provided using a regional protocol that incorporates the standards and guidelines of the European Resuscitation Council (Antwerp, Belgium).

Exclusion criteria of the study were documented terminal illness, successful defibrillation without administration of a vasopressor, and severe hypothermia (< 30°C). We compared pet_CO2 _and MAP in two treatment groups of resuscitated OHCA patients. In the epinephrine group, patients received 1 mg of epinephrine intravenously every three minutes. In the vasopressin/epinephrine group, patients received 40 units of arginine vasopressin (Pitressin; Goldshield Pharmaceuticals Ltd, Surrey, UK) intravenously only or followed by 1 mg of epinephrine every three minutes during CPR. Patient allocation into these two groups depended on the year of incident (vasopressin has been the first therapy in ventricular fibrillation since November 2003 and in asystole since January 2005) and on accessibility of vasopressin in our prehospital unit (intermittently available since November 2000 and regularly available since November 2003). After successful resuscitation, patients were transferred to the intensive care unit of the Teaching Hospital Maribor.

Data were collected and analyzed according to the Utstein criteria. Demographic information, medical data, and pet_CO2 _values were recorded for each patient by the emergency physician.

During resuscitation, the pet_CO2 _values were measured and recorded every minute beginning with the initial postintubation pet_CO2 _(first pet_CO2 _value obtained) and ending with the final pet_CO2 _value at admission to the hospital. The initial MAP was the first measurement of MAP after ROSC, and the final MAP was recorded at admission to the hospital. Measurements of pet_CO2_, arterial blood pressure, and other parameters were performed with a LIFEPACK 12 defibrillator monitor (Physio-Control, Inc., part of Medtronic, Inc., Minneapolis, MN, USA). Hospital records were used for outcome analysis, including assessment of cerebral performance category (CPC), for patients discharged alive.

Data were expressed as mean ± standard deviation or as number (percentage). For analysis of variables, we used the Fisher exact test and the Wilcoxon rank sum test. The Bonferroni correction was applied for multiple comparisons. The null hypothesis was considered to be rejected at *p *values of less than 0.05. Analyses of independent predictors for ROSC and survival from univariate analysis were performed using a multivariate logistic regression. For statistical analysis, we used SPSS software (version 12.01; SPSS Inc., Chicago, IL, USA).

## Results

Out of 636 patients, 38 were excluded from the study because they were successfully defibrillated without administration of a vasopressor. Patients from the recent vasopressin study [6] (patients with initial ventricular fibrillation) were included in this study. There were no significant differences between demographic and initial clinical characteristics in the compared groups: first monitored rhythm, location of arrest, witnessed arrest, etiology of arrest, gender, age, time to initiation of CPR, and initial pet_CO2 _(Tables 1 to 3).

**Table 1:** Utstein reporting for cardiopulmonary resuscitation in epinephrine and vasopressin groups

CPR data	Epinephrine group	Vasopressin group	*p *value^a^
Resuscitation attempts	*n *= 452	*n *= 146	
First monitored rhythm			
Shockable	175/452 (39%)	70/146 (48%)	0.22
VF	153/452 (34%)	62/146 (42%)	
VT	22/452 (5%)	8/146 (5%)	
Non-shockable	277/452 (61%)	76/146 (52%)	0.28
Asystole	183/452 (40%)	44/146 (30%)	
PEA	94/452 (21%)	32/146 (22%)	
Location of arrest			
Home	235/452 (52%)	76/146 (52%)	0.91
Public place	172/452 (38%)	55/146 (38%)	
Other	45/452 (10%)	15/146 (10%)	
Arrest witnessed			
By lay persons	277/452 (61%)	98/146 (67%)	0.67
By health care personnel	40/452 (9%)	16/146 (11%)	
Arrest not witnessed	135/452 (30%)	32/146 (22%)	
Etiology			
Presumed cardiac	307/452 (68%)	98/146 (67%)	0.69
Trauma	10/452 (2%)	7/146 (5%)	
Submersion	21/452 (5%)	7/146 (5%)	
Respiratory	41/452 (9%)	15/146 (10%)	
Other non-cardiac	36/452 (8%)	11/146 (8%)	
Unknown	37/452 (8%)	8/146 (6%)	
Outcome (number)			
Any ROSC	262/452 (58%)	98/146 (67%)	0.04
ROSC and admission to hospital	207/452 (46%)	91/146 (62%)	0.01
Survived 24 hours	157/452 (35%)	75/146 (51%)	0.02
Discharged alive	90/452 (20%)	36/146 (25%)	0.19

^a^By Fisher exact test. CPR, cardiopulmonary resuscitation; PEA, pulseless electrical activity; ROSC, restoration of spontaneous circulation; VF, ventricular fibrillation; VT, ventricular tachycardia without pulse.

**Table 2:** Demographic and clinical characteristics of out-of-hospital cardiac arrest patients

Characteristics	Epinephrine group (*n *= 452)	Vasopressin group (*n *= 146)
Males/females^a^	301/151	95/51
Age in years^b^	62.2 ± 17.8	60.8 ± 15.9
Bystander CPR, number (percentage)^a^	99/452 (22%)	31/146 (21%)
Time to initiation of CPR in minutes^b^	8.6 ± 5.3	7.8 ± 5.1
Average dose of epinephrine in milligrams^b,c^	7.6 ± 4.2	4.5 ± 2.7
Bicarbonate, number (percentage)^a,c^	172/452 (38%)	31/146 (21%)
Atropine, number (percentage)^a,c^	186/452 (41%)	42/146 (29%)
Dopamine, dobutamine, and norepinephrine, number (percentage)^a,c^	98/452 (22%)	15/146 (10%)
Resuscitation by medical team in minutes^b,c^	29.3 ± 9.4	18.7 ± 7.8

^a^By Fisher exact test; ^b^by Wilcoxon rank sum test; ^c^*p *< 0.05. CPR, cardiopulmonary resuscitation.

**Table 3:** End-tidal pressure of carbon dioxide and mean arterial blood pressure in two groups of cardiac arrest patients

Variables	Epinephrine group	Vasopressin group	*p *value^a^
Median of pet_CO2 _reading	16	15	0.86
Interquartile range	5–26	6–23	
Average pet_CO2 _(patients with ROSC)	2.12 ± 0.51	3.6 ± 0.86	< 0.01
Average pet_CO2 _(patients without ROSC)	0.92 ± 0.28	1.78 ± 0.58	< 0.01
Initial pet_CO2 _(patients with ROSC)	2.24 ± 0.81	2.13 ± 0.72	0.87
Initial pet_CO2 _(patients without ROSC)	0.85 ± 0.64	1.05 ± 0.64	0.48
Final pet_CO2 _(patients with ROSC)	2.95 ± 0.42	4.68 ± 1.1	< 0.01
Final pet_CO2 _(patients without ROSC)	0.78 ± 0.52	0.88 ± 0.38	0.84
Average initial MAP	74.6 ± 11.3	92.4 ± 9.7	< 0.01
Average final MAP	80.3 ± 12.4	105.8 ± 16.1	< 0.01

^a^By Wilcoxon rank sum test. MAP, mean arterial blood pressure (in millimeters of mercury); pet_CO2_, end-tidal pressure of carbon dioxide (in kilopascals); ROSC, restoration of spontaneous circulation.

The initial, average, and final values of pet_CO2 _were significantly higher in patients with ROSC on admission to the hospital compared with patients without ROSC in both groups (*p *< 0.01). All patients with ROSC had an initial pet_CO2 _value greater than 1.33 kPa. The average pet_CO2 _values in patients with and without ROSC and the final pet_CO2 _values in patients with ROSC were significantly higher in the vasopressin/epinephrine group (*p *< 0.01). The average values of initial and final MAP were significantly higher in the vasopressin/epinephrine group (*p *< 0.01) (Table 3).

In multivariate analysis, initial, average, and final pet_CO2 _values, initial MAP, and use of vasopressin were associated with ROSC and admission to the hospital (Table 4); initial, average, and final pet_CO2 _values, final MAP, and use of vasopressin were associated with survival at 24 hours (Table 5); initial, average, and final pet_CO2 _values and final MAP were associated with final survival and hospital discharge (Table 6). Vasopressin/epinephrine therapy was not associated with improved hospital discharge.

**Table 4:** Variables associated with restoration of spontaneous circulation and hospital admission

Variables	Odds ratio	95% confidence interval	*p *value
Shockable rhythm (VF, VT)	2.11	1.14–2.87	0.016
Arrival time	1.38^a^	1.07–2.55	0.008
Witnessed arrest	1.27	0.76–1.94	0.54
Bystander CPR	2.43	1.21–4.98	0.014
Initial pet_CO2 _^b^	20.35	5.43–35.63	<0.001
Average pet_CO2 _^b^	6.36	2.30–8.34	<0.001
Final pet_CO2 _^b^	2.85	1.43–3.92	0.003
Initial MAP^b^	1.25	1.13–1.86	0.02
Vasopressin	1.63	1.24–2.14	0.012
Gender (female)	2.85	1.36–5.48	0.002
Period 2^c^	1.28	1.15–1.92	0.02

^a^Value proportional to each one-minute decrease in arrival time; ^b^Values proportional to each increase by 1.33 kPa (10 mm Hg); ^c^CPR performed in the period from November 2003 to April 2006 (period 1: January 2000 to November 2003). CPR, cardiopulmonary resuscitation; MAP, mean arterial blood pressure; pet_CO2_, end-tidal pressure of carbon dioxide; VF, ventricular fibrillation; VT, ventricular tachycardia without pulse.

**Table 5:** Variables associated with survival at 24 hours

Variables	Odds ratio	95% confidence interval	*p *value
Shockable rhythm (VF, VT)	1.27	1.08–1.58	0.02
Arrival time	1.32^a^	1.24–1.68	0.01
Witnessed arrest	7.64	2.32–22.42	< 0.001
Bystander CPR	4.84	2.10–10.48	< 0.001
Initial pet_CO2 _^b^	18.65	6.14–32.27	< 0.001
Average pet_CO2 _^b^	2.86	1.42–4.65	< 0.001
Final pet_CO2 _^b^	2.26	1.21–4.13	0.012
Initial MAP^b^	1.06	0.82–1.43	0.46
Final MAP^b^	1.79	1.28–3.12	0.009
Vasopressin	1.34	1.14–1.94	0.024
Period 2^c^	1.68	1.20–2.94	0.008

^a^Value proportional to each one-minute decrease in arrival time; ^b^Values proportional to each increase by 1.33 kPa (10 mm Hg); ^c^CPR performed in the period from November 2003 to April 2006 (period 1: January 2000 to November 2003). CPR, cardiopulmonary resuscitation; MAP, mean arterial blood pressure; pet_CO2_, end-tidal pressure of carbon dioxide.

**Table 6:** Variables associated with hospital discharge

Variables	Odds ratio	95% confidence interval	*p *value
Shockable rhythm (VF, VT)	1.34	1.22–1.92	0.03
Arrival time	1.46^a^	1.26–2.12	0.01
Witnessed arrest	6.84	2.27–20.67	< 0.001
Bystander CPR	4.45	1.98–9.48	< 0.001
Initial pet_CO2 _^b^	1.61	1.28–2.64	0.008
Average pet_CO2 _^b^	1.47	1.22–1.93	0.014
Final pet_CO2 _^b^	2.67	1.83–3.68	< 0.001
Initial MAP^b^	1.02	0.91–1.32	0.54
Final MAP^b^	1.39	1.23–2.13	0.01
Vasopressin	1.12	0.82–1.33	0.42
Period 2^c^	1.32	1.19–1.95	0.03

^a^Value proportional to each one-minute decrease in arrival time; ^b^Values proportional to each increase by 1.33 kPa (10 mm Hg); ^c^CPR performed in the period from November 2003 to April 2006 (period 1: January 2000 to November 2003). CPR, cardiopulmonary resuscitation; MAP, mean arterial blood pressure; pet_CO2_, end-tidal pressure of carbon dioxide.

In outcome analysis, we found significantly higher rates of ROSC and survival at 24 hours in the vasopressin/epinephrine group (*p *< 0.05) (Table 1). There was no difference in survival to hospital discharge between groups (*p *= 0.19), but when analyzing the subgroup of patients in asystole, we found a significantly higher hospital discharge rate in patients treated with vasopressin (epinephrine subgroup 17/183 [9.3%] versus vasopressin/epinephrine subgroup 10/44 [22.7%]; *p *= 0.04; Fisher exact test). In the epinephrine group, significantly higher doses of additional epinephrine were needed, CPR lasted longer, and significantly more patients needed additional atropine, bicarbonate, and inotropic agents than in the vasopressin/epinephrine group (*p *< 0.05).

Out of all cases of cardiac arrest, 90 patients in the epinephrine group and 36 in the vasopressin/epinephrine group were discharged alive from the hospital. Forty-seven discharged patients in the epinephrine group were with CPC-1 or CPC-2 (52% of survivors), 37 patients with CPC-3 or CPC-4 (41%), and 6 patients with CPC-5 (7%). In the vasopressin/epinephrine group, 26 discharged patients were with CPC-1 or CPC-2 (72%), 8 patients with CPC-3 or CPC-4 (22%), and 2 patients with CPC-5 (6%). Neurological outcome of discharged patients was better (CPC-1 or CPC-2) in the vasopressin/epinephrine group (*p *= 0.04).

## Discussion

In previous studies, the relationship between pet_CO2 _and prognosis was established in prehospital CPR [11-13]. In this study, however, the main focus was on the relationship between pet_CO2 _and MAP and subsequent outcomes. The relevant hemodynamic parameters of resuscitated patients treated with epinephrine only and patients treated with vasopressin (only or in combination with epinephrine) were compared along with their prognostic value in CPR outcome.

The results of this study are similar to those of the studies of Wenzel and colleagues [4] and Guyette and colleagues [5] and show higher rates of ROSC and survival at 24 hours in the group of patients treated with vasopressin. In addition, this study shows that the patients who had asystole as the initial arrest rhythm and who were treated with vasopressin have a higher hospital discharge rate. The average and final pet_CO2 _values in vasopressin-treated patients with ROSC were significantly higher. The initial and the final MAP values were significantly higher in the vasopressin group as well. These results suggest that vasopressin could be more potent than epinephrine in increasing the cardiac output.

Using an animal model, Isserles and Breen [16] established a linear relationship between changes in pet_CO2 _and cardiac output. The authors claim that, during a decreased cardiac output, reduced carbon dioxide delivery to the lung decreases alveolar carbon dioxide pressure and thus causes part of the decrease in pet_CO2_. The remaining reduction in pet_CO2 _results from the increase in alveolar dead space due to the lower pulmonary perfusion pressure (dilution of carbon dioxide from perfused alveolar spaces). Gazmuri and colleagues [17,18] confirmed that both pet_CO2 _and Pa_CO2 _(arterial partial pressure of carbon dioxide) correspond with the pulmonary blood flow and therefore with cardiac output generated by precordial compressions during CPR.

In an animal study, Yannopoulos and colleagues [19] demonstrated a linear correlation between MAP, cerebral perfusion pressure and CPP, and pet_CO2_. A strong correlation between MAP and neurological outcome was observed in a few other studies [20-22]. In a study using a pig model of ventricular fibrillation cardiac arrest, Lindner and colleagues [23] concluded that administration of vasopressin led to a significantly higher CPP, myocardial blood flow, and total cerebral flow during CPR. In a study conducted by Morris and colleagues [24] using a human model of prolonged cardiac arrest, 40% of the patients receiving vasopressin had a significant increase in CPP. Our study shows that higher values of pet_CO2 _and MAP in patients treated with vasopressin are consistent with the better outcomes in the vasopressin group. In a multivariate analysis, we determined that the chances for survival are improved in patients with a higher MAP on admission to the hospital (for every 1.33-kPa increase in MAP, the chances for survival were 1.4 times better). We also determined that the chances for ROSC, survival at 24 hours, and hospital discharge are associated with the year in which CPR was administered (Tables 4 to 6). Various factors may cause differences between the two observed time periods. These include implementation of new CPR guidelines, renewal of dispatch protocols, application of vasopressin as first therapy, and improved phone communication.

In our study, we had significantly more patients with CPC-1 and CPC-2 in the vasopressin group than in the epinephrine group. In the postresuscitation period, MAP is usually kept at a normal level (80 to 100 mm Hg) or at least at a level that secures coronary perfusion (that is, 65 mm Hg). Results from the study by Bell and colleagues [25] indicate that, to secure cerebral perfusion and prevent secondary cerebral injury, MAP should be kept at a level higher than commonly accepted. In our study, vasopressin contributed to a higher average final MAP (approximately 105 mm Hg), thus preserving cerebral perfusion in the critical postresuscitation period of absent cerebral autoregulation.

Several investigations have demonstrated that vasopressin could improve hemodynamic variables in advanced vasodilatatory or hemorrhagic shock [26-32]. The study by Friesenecker and colleagues [27] showed that, under normal physiological conditions, vasopressin exerted significantly stronger vasoconstriction on large arterioles than norepinephrine. This observation could explain, in part, why vasopressin can be effective in advanced shock that is unresponsive to increases of catecholamines in the standard shock therapy.

In the epinephrine group, resuscitation efforts lasted longer and a significantly higher quantity of additional epinephrine was needed. Adrenergic stimulation by additional doses of epinephrine is associated with adverse cardiac effects, including postresuscitation myocardial dysfunction and increased myocardial oxygen consumption. That is one of the reasons why significantly larger doses of additional therapy (inotropes, vasopressors, atropine, and bicarbonate) were needed in the epinephrine group in comparison with the vasopressin group.

Increased doses of epinephrine have a direct impact on lowering the pet_CO2 _value [33]. Tang and colleagues [34] in an experimental model and Cantineau and colleagues [35] in a prospective human study established that epinephrine induces pulmonary ventilation/perfusion defects as a result of redistribution of pulmonary blood flow. Other studies show that high doses of epinephrine significantly decrease cardiac output and pet_CO2 _but enhance myocardial perfusion pressure and myocardial blood flow [36,37]. Lindberg and colleagues [38] confirmed that an injection of epinephrine during chest compressions decreased pet_CO2 _and pulmonary blood flow and increased CPP (which then slowly decreased), but the effects on pet_CO2 _and pulmonary blood flow were prolonged. Therefore, epinephrine initially increases CPP and the chances of ROSC, but decreases pet_CO2 _value induced by critical deterioration in cardiac output and thereby diminishes oxygen delivery.

Tang and colleagues [39] confirmed that the beta-adrenergic action of epinephrine has a detrimental effect on postresuscitation myocardial function because it increases myocardial oxygen consumption and decreases postresuscitation survival. In the study by Pan and colleagues [40], CPP was increased after vasopressin application and a significant positive correlation between pet_CO2 _and CPP was observed, suggesting that vasopressin has very little effect on pulmonary circulation and ventilation/perfusion mismatch.

Unlike vasopressin, epinephrine during CPR can, to some extent, reduce pet_CO2 _values because of its impact on the pulmonary circulation. Nevertheless, the values of pet_CO2_, together with MAP, reliably reflect changes in cardiac output.

## Conclusion

Pet_CO2 _and MAP values are prognostic factors for the outcome of OHCA. During a cardiac arrest, pet_CO2 _can be considered an indirect parameter for the evaluation of cardiac output in prehospital monitoring together with MAP, when spontaneous circulation is restored. Patients treated with vasopressin alone or followed by epinephrine during CPR have higher average and final pet_CO2 _values as well as higher initial and final MAP values on admission to the hospital than patients treated with epinephrine only. The combination of vasopressor therapy (vasopressin followed by epinephrine) in CPR improves ROSC as well as short-term survival and neurological outcome. In the subgroup of patients with asystole as the initial rhythm, it improves the hospital discharge rate. Our findings suggest that the current guidelines for resuscitation established by the European Resuscitation Council, in which vasopressin is not considered even as a secondary alternative to epinephrine, should be revised.

## Key messages

• During CPR, higher pet_CO2 _and MAP values were observed when vasopressin was used.

• Pet_CO2 _and MAP are strong prognostic factors for the outcome of cardiac arrest.

• Compared to epinephrine, vasopressin in CPR improves ROSC as well as short-term survival and neurological outcome.

## Abbreviations

ALS = advanced life support; CPC = cerebral performance category; CPP = coronary perfusion pressure; CPR = cardiopulmonary resuscitation; MAP = mean arterial blood pressure; OHCA = out-of-hospital cardiac arrest; pet_CO2 _= end-tidal carbon dioxide tension; ROSC = restoration of spontaneous circulation.

## Competing interests

The authors declare that they have no competing interests.

## Authors' contributions

SM participated in conceiving and designing the study and drafted the manuscript. AJ participated in collecting data and helped to draft the manuscript. SG performed the statistical analysis and made critical revisions of the study. All authors have read and approved the final manuscript.
